# Supplementary material for: Abrupt events and population synchrony in the dynamics of Bovine Tuberculosis
Source: Nat Commun. 2018 Jul 19;9:2821. doi: 10.1038/s41467-018-04915-0 (PMC6053421; doi:10.1038/s41467-018-04915-0)
Supplement: Supplementary file 1 — Supplementary Information [file 41467_2018_4915_MOESM1_ESM.pdf]

Supplementary Information

Abrupt events and population synchrony in the dynamics of *Bovine Tuberculosis*

Moustakas et al.

Supplementary Note 1 – Seasonality of NHI/TTH

For all the analyses, NHI (new herd incidents) were divided by TTH (total tests on herds); NHI/TTH. This accounts for the fact that (a) higher NHI can be found at locations with higher number of total herds, or with more frequent testing, (b) herds not bTB free are revisited for testing more often, and (c) there is a within-year seasonality in testing with generally more tests carried out during winter months. Therefore, purposive sampling is employed [1]. In order to examine whether NHI/TTH is a seasonally unbiased estimator of bTB spread, the residuals per month of a simple (assuming a single linear trend and not accounting for stationarity) time series decomposition was performed.

Time series decomposition separates the time series into linear trend and seasonal components, as well as error and stochastic fluctuations. As the seasonal pattern in the data depended on the level of the data—more cattle tests are carried out during winter months and in general as more tests are carried out, more TB infected herds are likely to be found [2]—a multiplicative model structure was employed accounting for this effect [3]. A multiplicative de-trending model is used when the size of the seasonal pattern in the data depends on the level of the data [3]. This analysis was used as a null model in order to examine whether the sensitivity of NHI/TTH changed within season because herds are tested more often during winter months or between years depending on testing intervals. The analysis indicated that the seasonality in the NHI/TTH index is negligible; all residuals by season which in our case is residuals per month (lower right graph in each panel; see also the red dotted reference line indicating zero) are not significantly different from each other (all 50% confidence intervals of the mean of residuals per month 1-12 do not differ from each other). This is applicable in the full GB dataset (a), as well as in specific locations where annual testing (b) or testing every four years (c) is applied. *This indicates that the NHI/TTH has negligible seasonality and that this is applicable in annual testing areas, in four year testing areas as*

well as in the full dataset as even a 'naïve' time series decomposition analysis produces residuals that are do not differ from each other based on a monthly basis (seasonal analysis within each year).

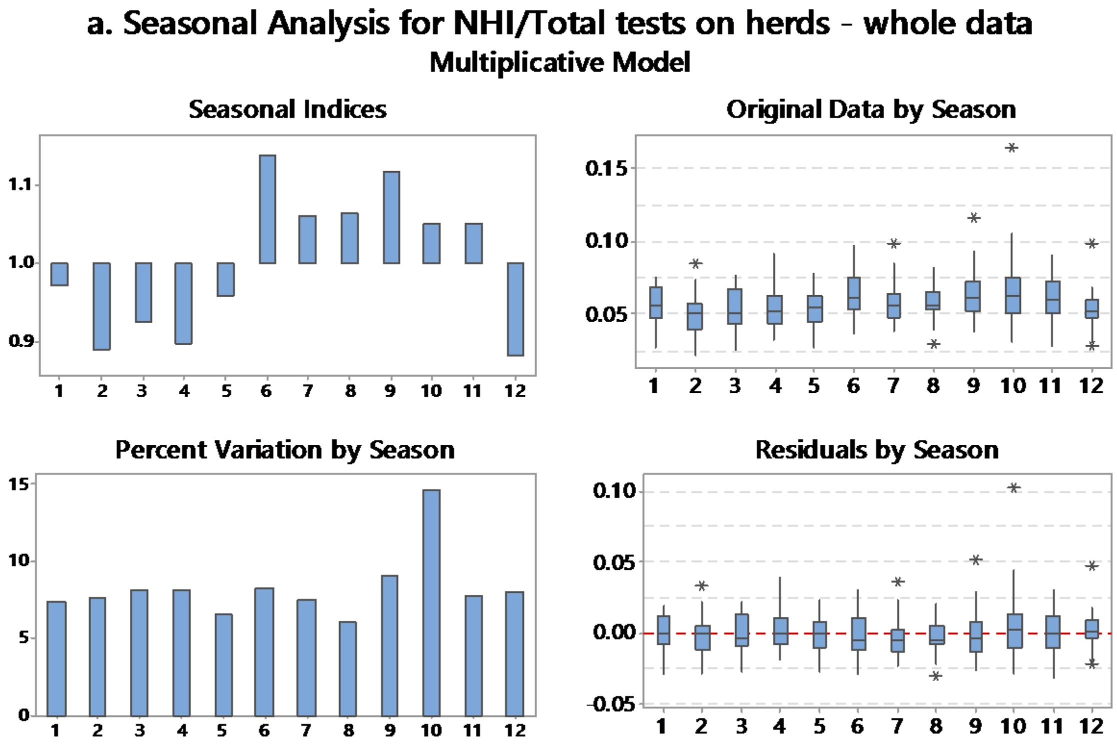

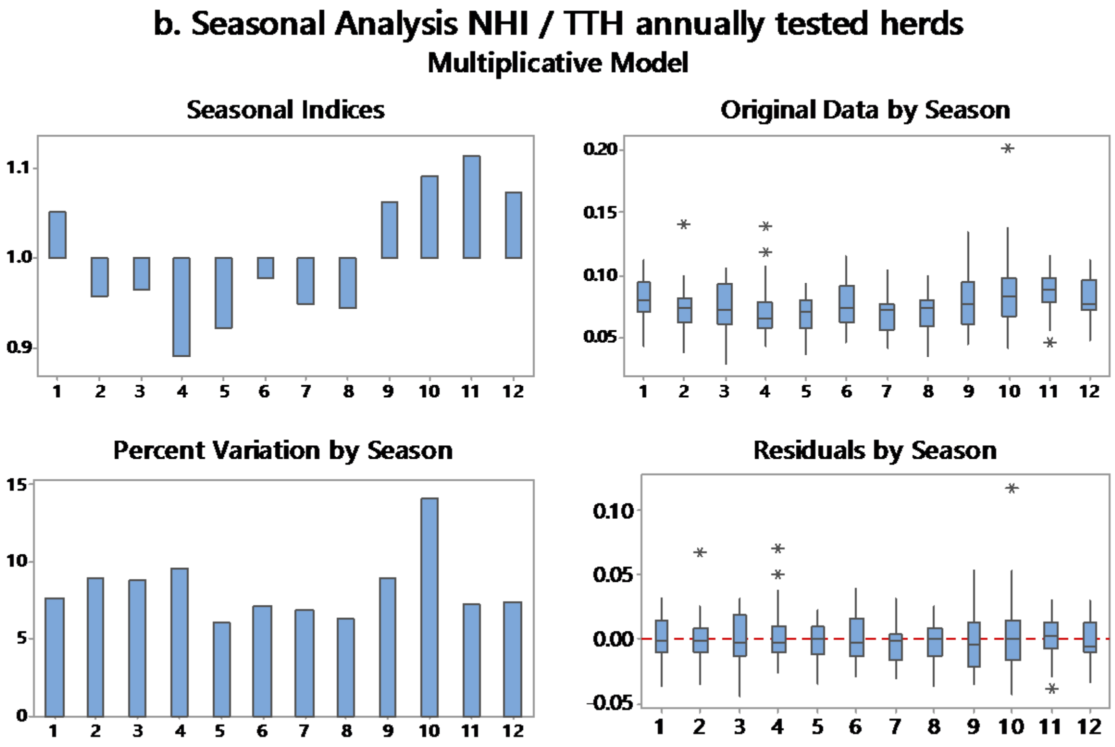

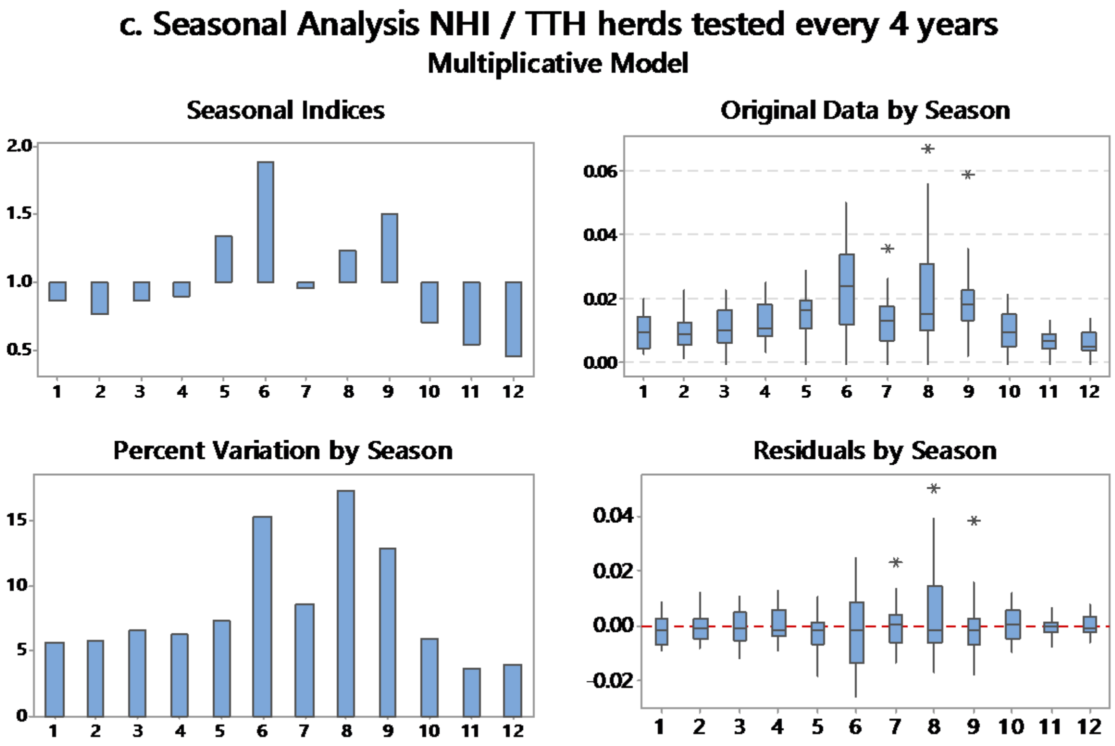

Supplementary Figure 1. Seasonal Analysis for NHI/TTH. **(a)** Analysis of seasonality of NHI/TTH of the full dataset **(b)** Analysis of seasonality of NHI/TTH of annually tested herds. **(c)** Analysis of

seasonality of NHI/TTH of herds tested every four years. All residuals by season (lower right panel graph in all three figures) indicate that there is negligible influence of seasonality in the NHI/TTH data as none of the confidence intervals of the mean (blue-coloured solid boxes) differ significantly from each other between months (all confidence intervals of the mean per month are within the confidence intervals of all other months means).

Supplementary Note 2. Probability distribution of NHI/TTH in Scotland

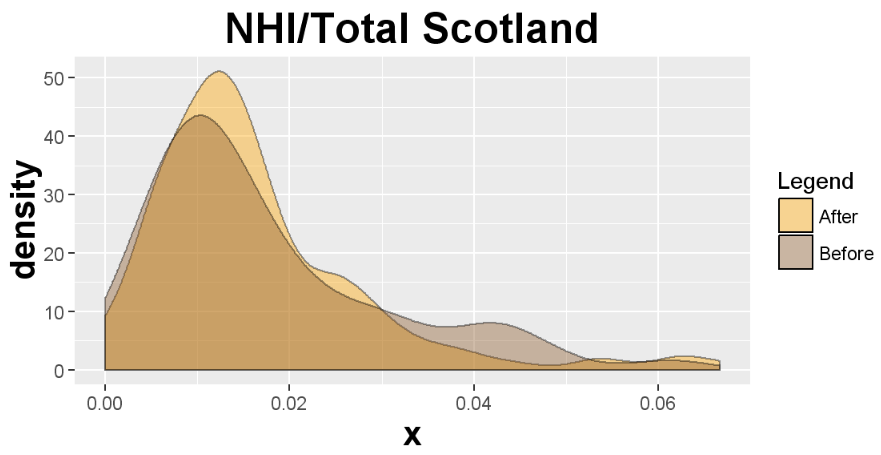

Supplementary Figure 2. Probability of NHI/TTH in Scotland. Empirical probability density plots of NHI/TTH in counties in Scotland before (dark brown colour) and after testing interruption (light brown colour). Testing interruption data are not included. The empirical probability distribution aggregated across all counties in Scotland was derived with the same procedure as the one described in the main text, Methods, *'Empirical Probability Distributions'*

Supplementary Note 3: Additional temporal analysis

Temporal autocorrelation analysis

We sought to quantify the temporal dynamics of disease spread in cattle before and after testing interruption in GB and in different regions of GB throughout the time span of the dataset. To do so we calculated the partial correlation function [4] for NHI/TTH of bTB twice, before and after testing interruption. Testing interruption months were discarded from the analysis. The analysis was performed using the 'acf' function in the 'stats' package in R [5]. Horizontal dotted lines in blue colour indicate 95% confidence intervals. The temporal autocorrelation analysis for GB as well as aggregated across GB regions:

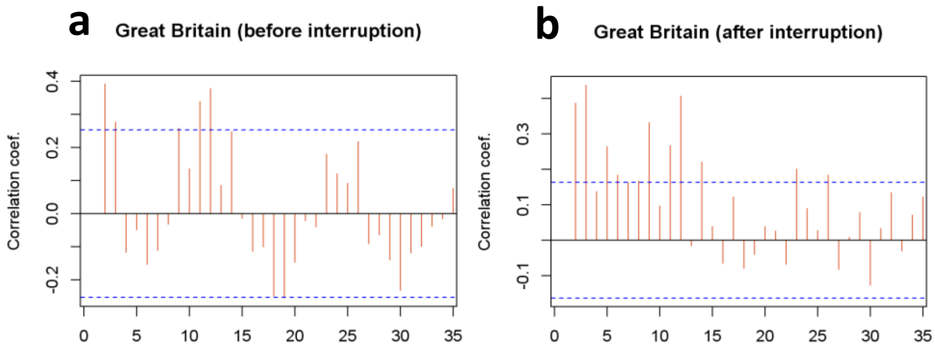

Supplementary Figure 3. Temporal autocorrelation analysis for GB. Temporal autocorrelation analysis for the full data before (a. left panel) and after (b. right panel) testing interruption (interruption data discarded). All time lag units (horizontal axis) are in months. Vertical axis indicates correlation values [-1, 1], with values close to one (positive or negative) indicating strong positive or negative correlation, while values close to zero indicate no correlation. Dotted horizontal lines indicate 95% significance.

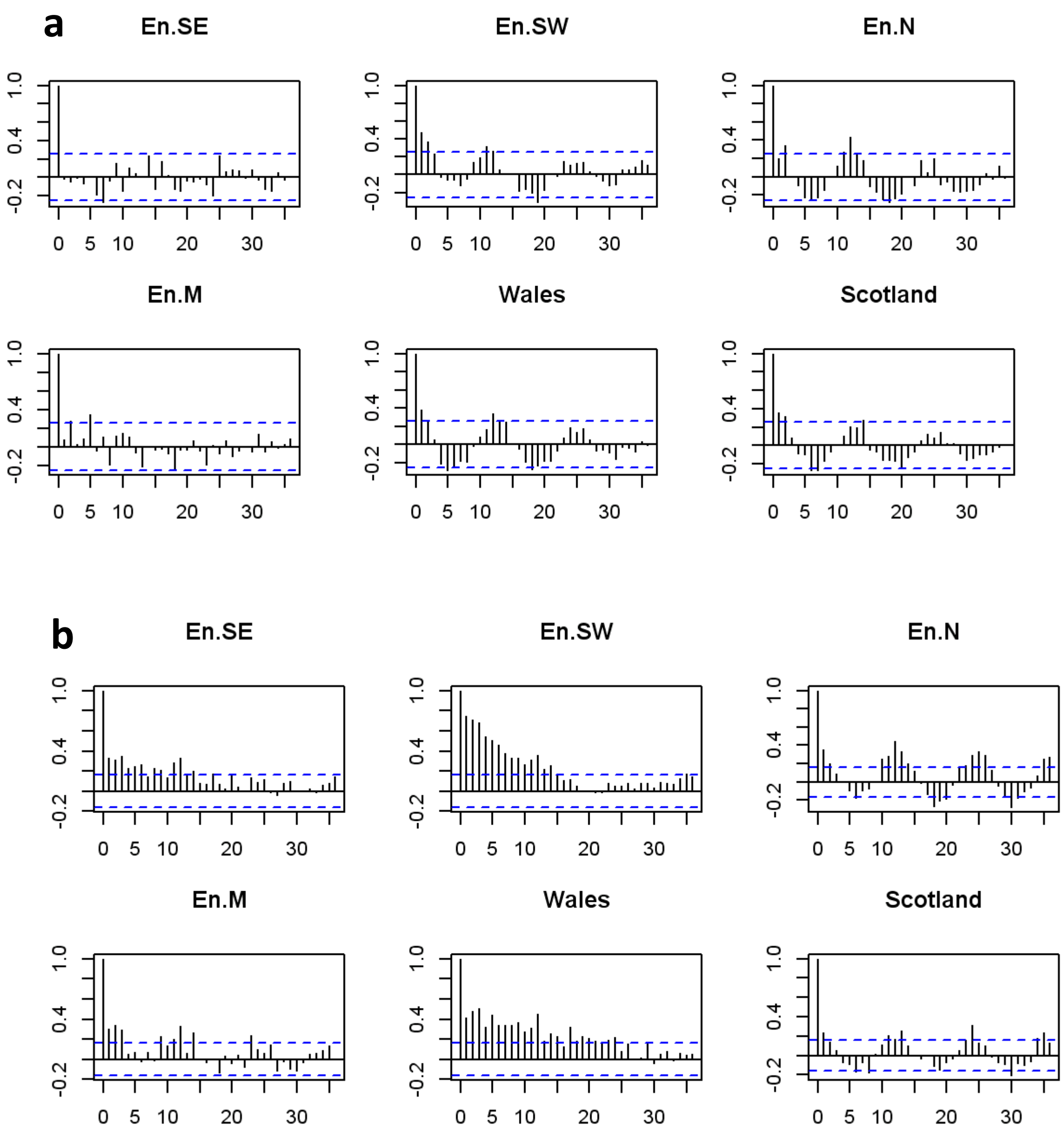

Supplementary Figure 4. Temporal autocorrelation analysis in different GB regions. Temporal autocorrelation analysis for the full data before (a. left panel) and after (b. right panel) testing interruption (interruption data discarded). All time lag units (horizontal axis) are in months. Vertical axis indicates correlation values [-1, 1], with values close to one (positive or negative) indicating strong positive or negative correlation, while values close to zero indicate no correlation. Dotted horizontal lines indicate 95% significance. (En: England, SE: South East, SW: South West, N: North, M: Midlands).

Temporal autocorrelation analysis of new infections indicated that the autocorrelation function changes dramatically after the interruption and that this is consistent for GB as well as for different regions in GB (Fig 1c, d in the main text as well as Supplementary Figure 3 and Supplementary Figure 4). The annual cycle is not the dominant fluctuation any more, being replaced by a more persistent behaviour, i.e. the autocorrelation coefficient maintains statistically significant values for larger monthly lags.

Wavelet energy of the mean time series.

We have followed the same methodology described in the main document in the Methods, section 'Wavelet analysis' to calculate periodicities estimated by wavelet analysis of the means (in the main document we list the mean field approach, we are replicating here with an alternative analysis using the means of time series, a method that is also commonly employed).

Wavelet analysis using the mean of the time series indicated that for GB for the entire period there was a strong and statistically significant cycle at around four years, at a 95% confidence level across all three significance wavelet tests [6, 7] (Supplementary Figure 5). The thick black contour designates statistical significance ($p > 0.95$) and the cone of influence, where edge effects can potentially distort the picture, is shown in a lighter shade [8, 9]. Note that not all the significant black contour needs to be within the cone of influence, i.e. if part of the significant contour falls within the cone of influence this still indicates a significant circle [9]. This cycle existed before the testing interruption; however, it is peaking during interruption period and eventually fading out during the introduction of at least annual testing in high risk areas of England in 2013 (Supplementary Figure 5a). Prior to testing interruption, there was an infection cycle with a 12-month period (Supplementary Figure 5b), whereas after testing interruption the infection cycle moved to 36-48 months with an underlying weaker 12-month cycle also persisting (Supplementary Figure 5c). This longer infection cycle (36-48 months) is still present in high risk areas (Supplementary Figure 5d), while in low risk areas the infection cycle did not persist after testing interruption, and a weak, non-significant, annual cycle is present (Supplementary Figure 5e). An analysis of the dataset with interruption data removed also confirmed the existence of an annual and a four-year cycle (Supplementary Figure 5f).

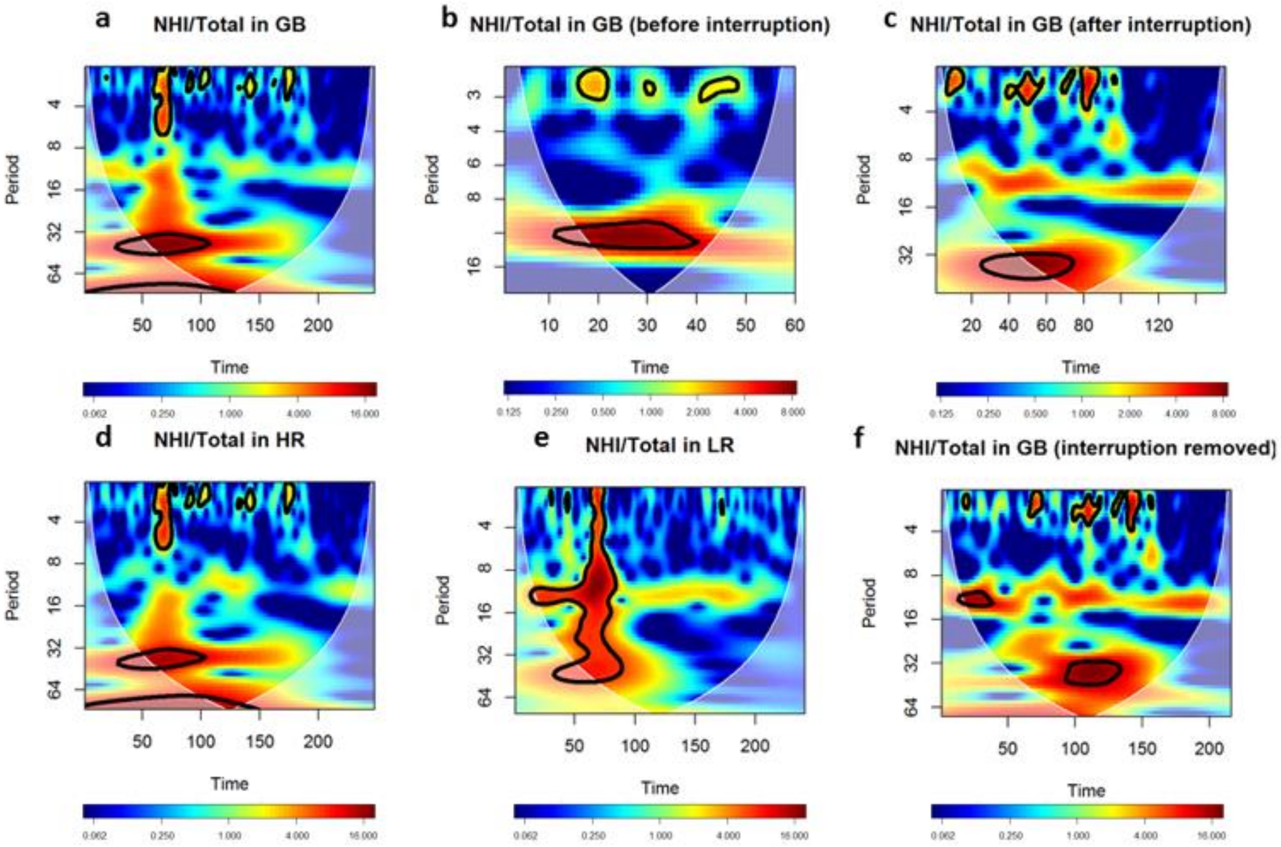

Supplementary Figure 5. Wavelets of the mean time series. Wavelet analysis of the mean time series of NHI/TTH data from January 1996 to August 2016. The vertical axis indicates periods (cycles) while the horizontal time. Both axes units are months. The thick black contour designates statistical significance ($p > 0.95$) and the cone of influence, where edge effects can potentially distort the picture, is shown as a lighter shade. **a.** GB for the whole period. **b.** GB before interruption, **c.** GB after interruption, **d.** High risk areas for the whole period and **e.** Low risk areas for the whole period. **f.** GB with interruption data removed.

Supplementary Note 4: Spatial synchrony and Network Analysis

Correlations matrices between counties in GB for each of the five time snapshots were estimated using the ‘pairwise.complete.obs’ in the ‘cor’ function in R [5] and plotted using the ‘print’ function with defined colour shading (deep blue shades indicate strong negative correlation, clear colour no correlation, deep red shades strong positive correlation). Each of the five correlation matrices (a – e) plotted here corresponds with the networks plotted in Figure 3 in the main document. The analysis and methodology is fully described in the main text, ‘Spatial synchrony and Network Analysis’ in the methods section.

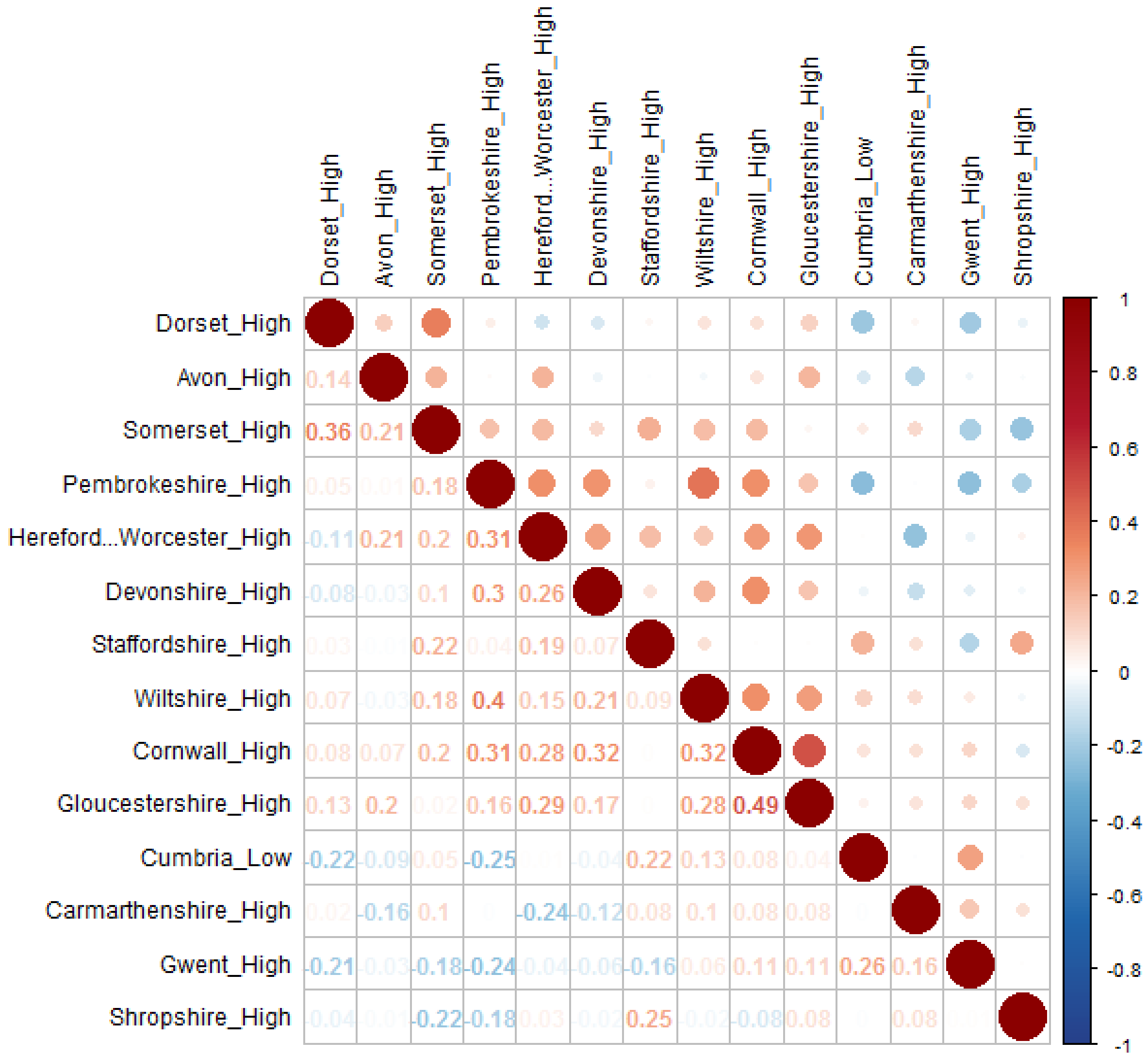
Dorset_High
Avon_High
Somerset_High
Pembrokeshire_High
Hereford...Worcester_High
Devonshire_High
Staffordshire_High
Wiltshire_High
Cornwall_High
Gloucestershire_High
Cumbria_Low
Carmarthenshire_High
Gwent_High
Shropshire_High
1
0.8
0.6
0.4
0.2
0
-0.2
-0.4
-0.6
-0.8
-1

a

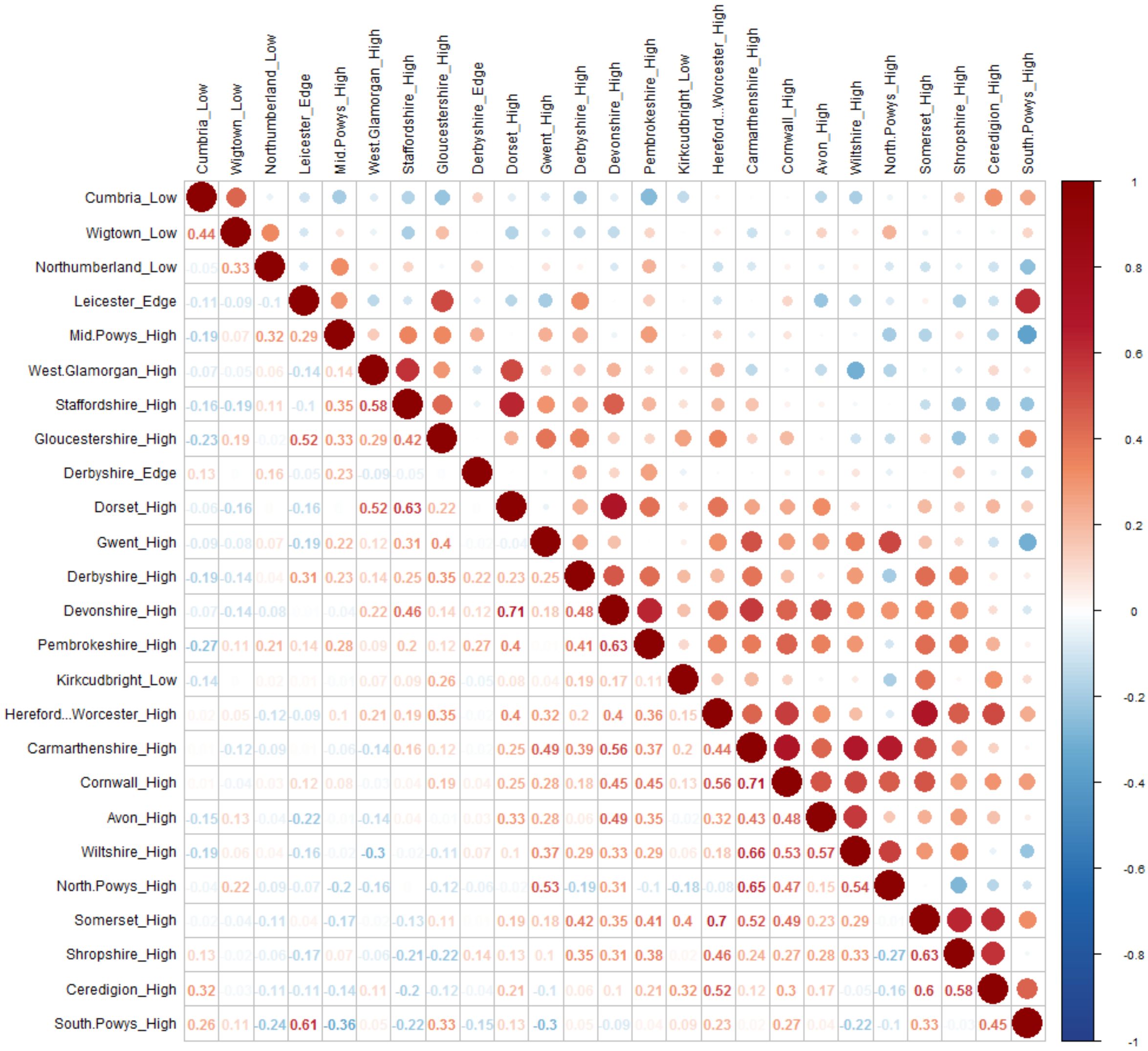

b

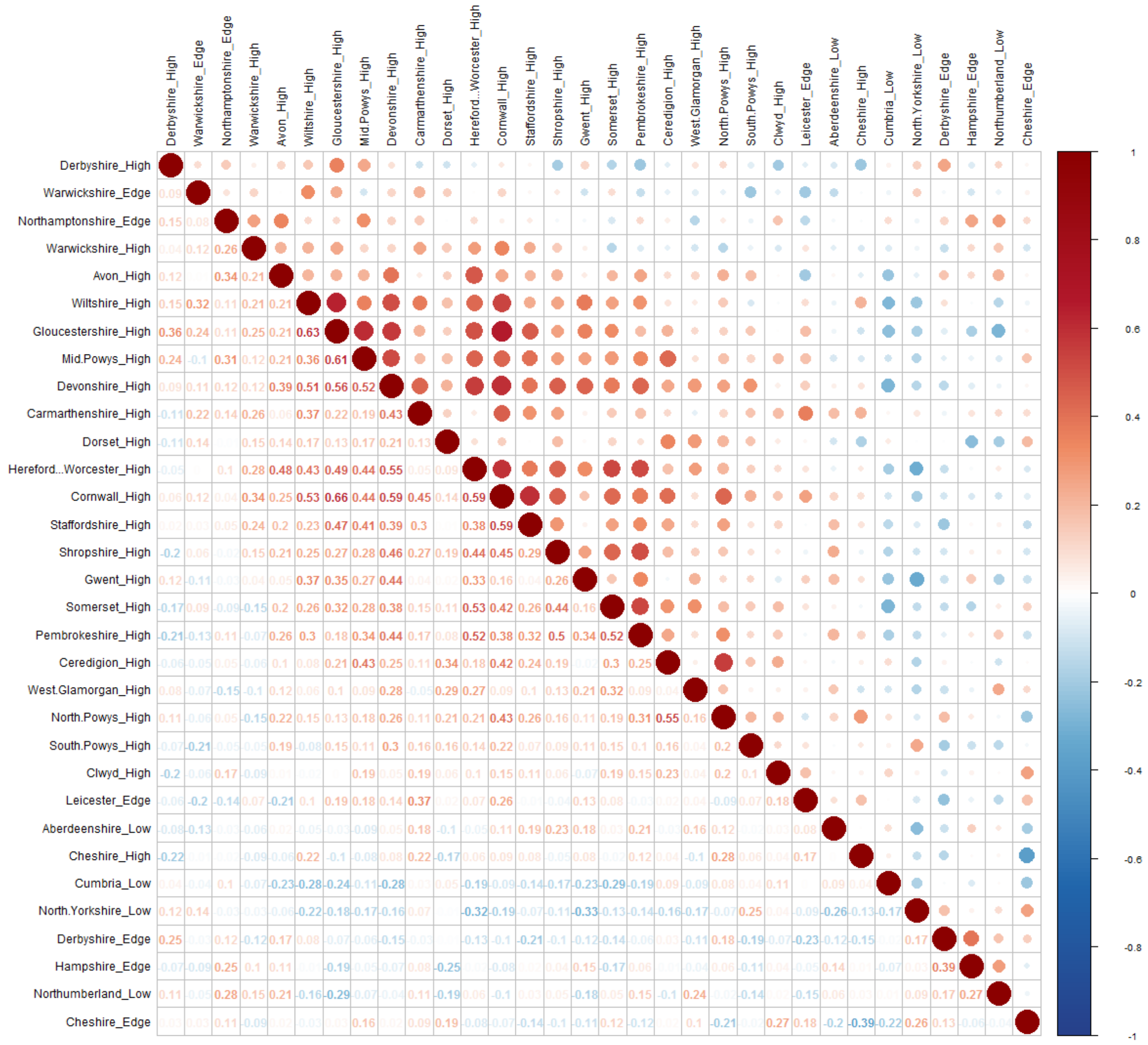

c

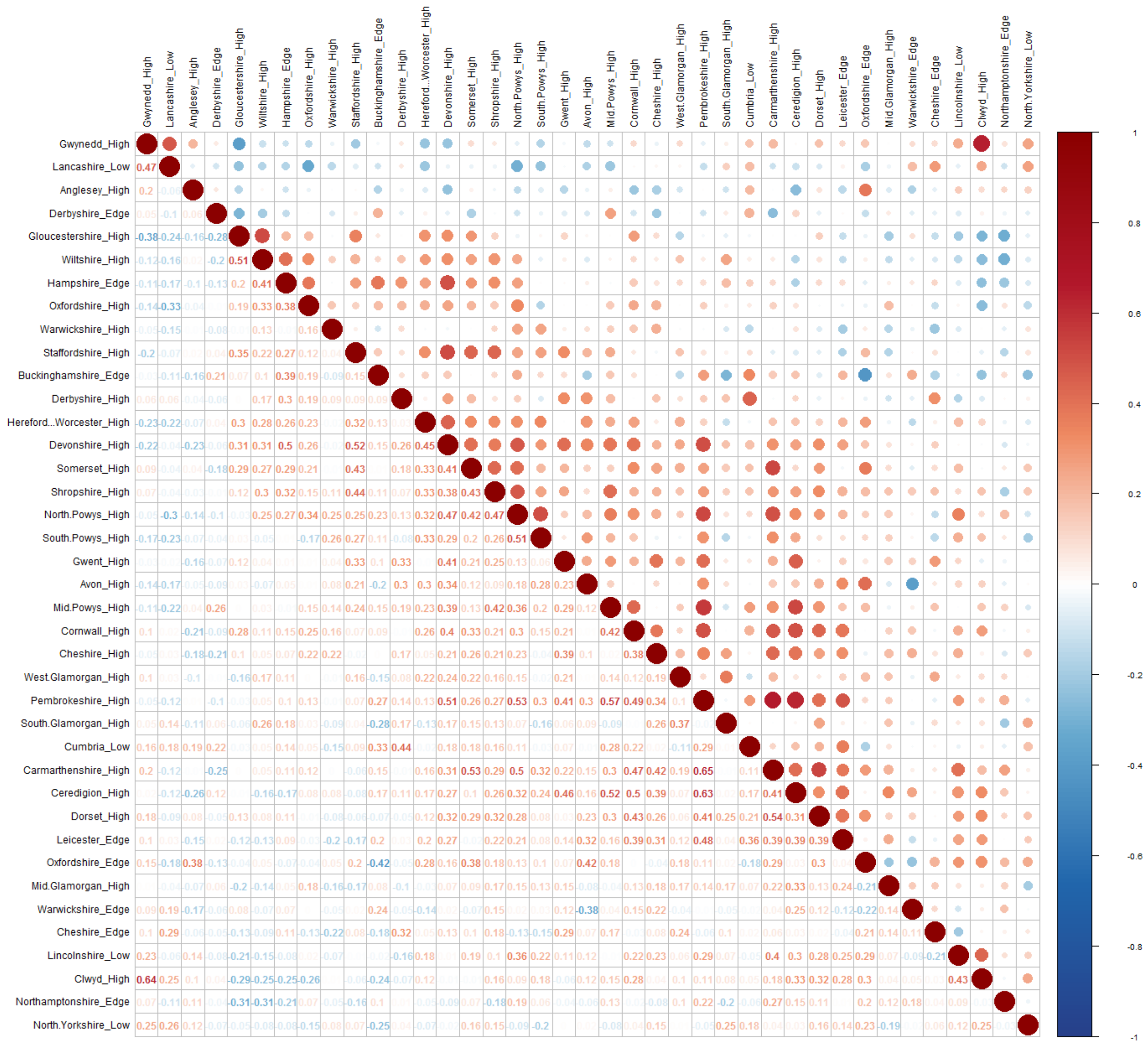

d

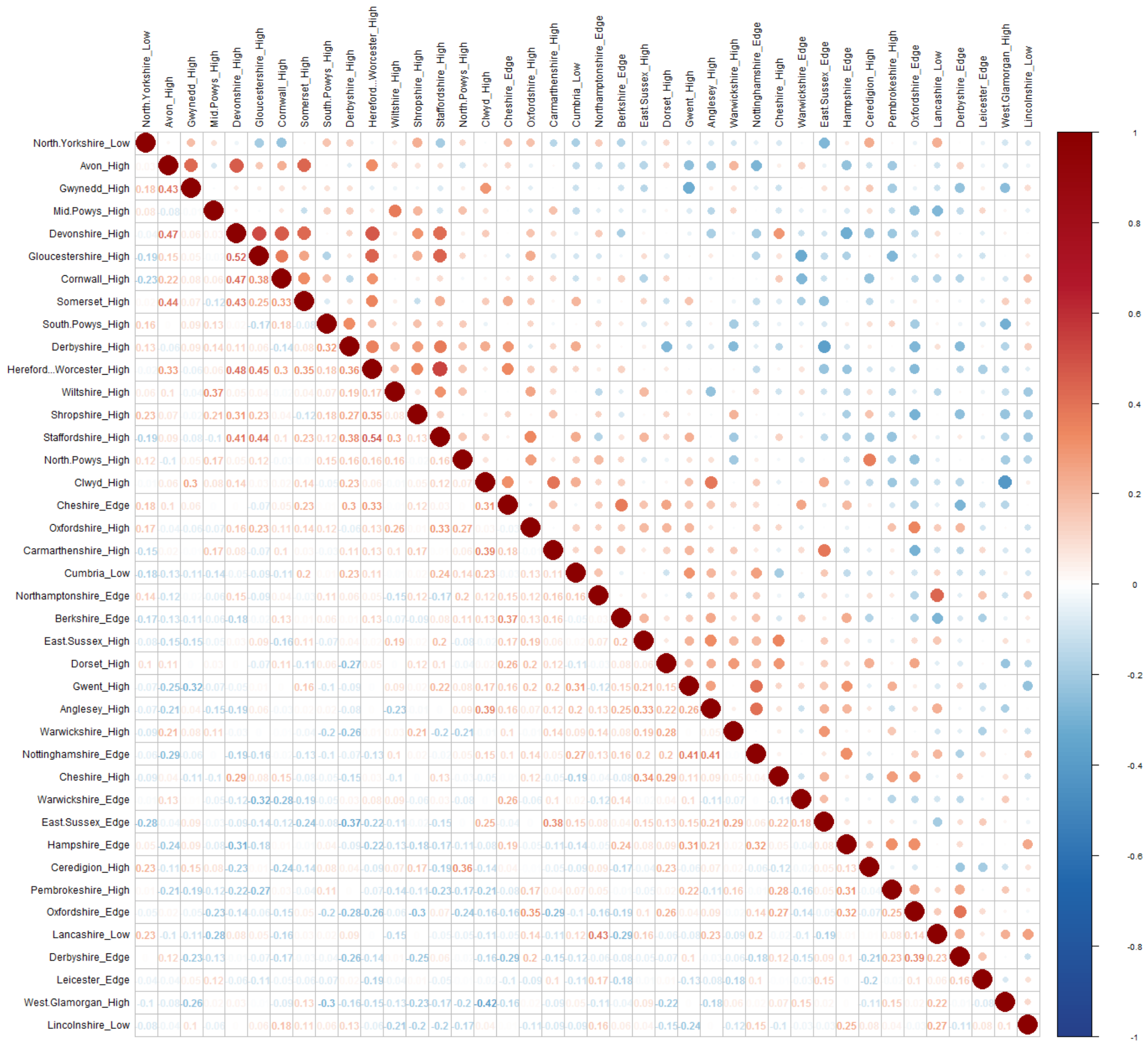

e

Supplementary Figure 6. Correlations matrices corresponding to Figure 3 of the main document. Correlation matrixes of differences in NHI/TTH per GB counties after the within the county annual variation has been removed. The numbers of nodes indicate the number of counties with high NHI/TTH values (counties above threshold), while the size of each bubble is proportional to the correlation value between those counties in the corresponding correlation matrix. Data are temporally partitioned into five snapshots as following: **(a)** 1/1996-3/2000, **(b)** 4/2000-4/2004, **(c)** 5/2004-6/2008, **(d)** 7/2008-7/2012, and **(e)** 8/2012-8/2016. The vertical red line indicates the introduction of annual testing in January 2013.

Supplementary Note 5: Individual Based Model (IBM)

Model description

A calibrated Individual Based Model (IBM) [6] of bTB was used to provide a potential mechanistic reproduction of the dynamics of bTB in cattle by having identical model parameters and varying only the frequency of cattle testing after testing interruption. The original model included both badgers and cattle, it has details of the behaviour and natural history of badgers and the husbandry of cattle as well as the dynamics of TB between and within species - see [10] for a full description. Here the model was used with cattle only (i.e. no badgers). In brief: the model is an ABM of cattle on a scale-specific grid for cattle. The cell size is 0.7 km^2. The grid size of cattle scales to the farm surface areas in the UK (four cells, 2.8 km^2; [11]). Cattle live on farms, give birth of one calf once a year, all the cattle on a farm are housed in one group (one cell of 0.7 km^2) over winter but spread around the farm in non-winter months (winter months = November to April; [12]), they live on average five years when healthy and up to two years when infected [13], cattle contract TB from each (cattle-to-cattle infection rate = 2.7%; [14]). In addition cattle are moved between farms [15] with mean distance that cattle moved throughout the UK 75.6 km yr^{-1} corresponding to 75.62 km yr^{-1} / 0.84 km $\approx$ 90 cells yr^{-1}(0.84 is the diagonal of a 0.7 km^2 cell). The standard deviation of the mean distance that cattle moved in the UK during those years was 84.13 km yr^{-1} corresponding to 84.13 km yr^{-1} / 0.84 km $\approx$ 100 cells yr^{-1}. The bTB test in cattle is imperfect i.e. it exhibits false negative cases as it is known to do [16]; (testing accuracy 70%). Cattle are tested for bTB prior to movement [9]. If a cattle individual tests positive, it is killed. The time step is one month. The model explicitly follows all cattle individuals. This model is run on a grid of 128 x 128 cells equivalent to the surface area of a large county in the UK [(128 x 128 cells, this is the simulation grid) x (0.7km^2 $cell^{-1}$, this is the cell size in the model)= 11,468.8 km^2, this is the total modelled surface area]. The duration of each simulation run is 360 months. The model is initialised with an initial number of cattle per unit area

scaling to 121 cattle km^{-2}, the mean cattle density in the UK [11]. Therefore 121 cattle km^{-2} x 0.7 km^{2} cell^{-1} x 128 x 128 cells = 1,387,725 cattle were simulated on each scenario. Each simulation scenario was replicated 10 times to account for model stochastic perturbations.

Model scenarios and parameterization

The introduction of annual testing in England in 2013

We initialised and run the IBM for high risk and edge areas in England, with parameter space identical to the one described in 'model description' above except the initial percentage of infected herds and testing frequency.

Scenario I

Testing frequency was set to every four years for the number of simulation months corresponding to post interruption period but before the introduction of annual testing from November 2002 to December 2012. For the number of simulation months corresponding from January 2013 to August 2016 annual testing frequency was applied as it happened in high risk and edge areas in England. Note that during bTB testing interruption period (January 2001 to October 2002) it is difficult to simulate the actual testing frequency; setting it to zero would imply no testing however herds were tested but not on a regular basis. The initial NHI/TTH ration was set to [(235 / 2698) x 100] = 8.7% corresponding to the mean of high risk areas in England in November 2002.

Scenario II

A second simulation scenario was performed was all lese been equal as described here but the bTB cattle testing interval did not change to annual testing; testing every four years throughout the simulation number of months corresponding from November 2002 to August 2016 was applied instead.

Quantifying the impact of testing frequency and testing interruption

Time series NHI/TTH data from high risk and edge areas in England were compared with the scenarios *I* and *II* IBM in terms of NHI/TTH outputs using the method 'Causal impact' a Bayesian inference time series intervention analysis [17]. Suppose that at time $t = \tau$ (where τ is known), there has been an intervention to a time series. By intervention, we mean a change to a procedure, such as the introduction of annual testing here, that we want to quantify this effect on the time series *x(t)*. We want to estimate how much the intervention has changed the series (if at all) [18]. When analyzing interventions through time series analysis we typically go through two steps:

(i) Finding a control time series for the test time series where the event took place using time series matching based on historical data prior to the event (the "pre period").

(ii) Analyzing the causal impact of the event by comparing the observed data for the response and control following the event (the "post period"), while factoring in differences between response and control data prior to the event.

Causal impact [17] is a novel time series intervention analysis method based on Bayesian inference. It is designed by scientists for big data analytics and it implements a Bayesian approach for estimating the causal effect of a designed intervention analysis on a time series. Given a response time series and a set of control time series, the method constructs a Bayesian structural time-series model. This model is then used to try and predict the counterfactual, i.e., how the response metric would have evolved after the intervention if the intervention had never occurred [17]. The underlying method employed in causal impact time series analysis allows inference of the temporal evolution of attributable impact, based on Bayesian empirical priors on the parameters, and can incorporate the time-varying influence of contemporaneous covariates [13]. The method uses a Markov chain Monte Carlo (MCMC) algorithm for model inversion. Causal impact is available in R [5] via the 'CausalImpact' package.

We sought to quantify the impact of the introduction of annual testing in high risk and edge areas in England in 2013 (intervention) by comparing data from these counties with IBM outputs. We first sought to quantify how sensitive is the model in incorporating this intervention and what is the deviance between model outputs and data. Hence the control time series are the data and the test time series is the one of model outputs. The time series variable analysed is NHI/TTH from November 2002 to August 2016). The same output time length was analysed from the IBM model. The intervention month is January 2013. In the IBM testing frequency switched to annual testing frequency in the same month (all else been equal), as it happened in the data. Therefore, the "intervention" here refers at quantifying the ability of the IBM to model the switch to annual testing – if everything was implemented correctly the intervention should have a minimal or no effect. These two time series were analysed with causal impact. During Causal impact analysis the arguments specifying seasonal components were set as number of seasons =12, and season duration = 1 (data are collected on a monthly basis, every year), and the default number of 1,000 MCMC inferences to draw was used.

Having quantified the potential deviance between data and IBM regarding bTB cattle testing frequency, all else been equal, we sought to quantify the impact of testing frequency the introduction. What would have happened if annual testing was not introduced in high risk and edge areas in 2013? To quantify this, the same data as in example 1 are compared with the IBM outputs all else been equal as in *scenario I* but with cattle testing frequency kept at four years throughout the simulation length. Thus the control time series are the data from *scenario II* and the test time series are the IBM outputs with cattle testing every four years throughout the simulation period.

Results - Figure 4a and 4b in the main document

Scenario I

During the post-intervention period (introduction of annual testing from Jan 2013 and onwards), the response variable had an average value of approx. 0.072. In the absence of an intervention, we would have expected an average response of 0.074. The 95% interval of this counterfactual prediction is [0.070, 0.077]. Subtracting this prediction from the observed response yields an estimate of the causal effect the intervention had on the response variable. This effect is -0.0011 with a 95% interval of [-0.0049, 0.0025].

Summing up the individual data points during the post-intervention period, the response variable had an overall value of 3.11. Had the intervention not taken place, we would have expected a sum of 3.16. The 95% interval of this prediction is [3.01, 3.33]. The above results are given in terms of absolute numbers. In relative terms, the response variable showed a decrease of-2%. The 95% interval of this percentage is [-7%, +3%].

This means that, although it may look as though the intervention has exerted a negative effect on the response variable when considering the intervention period as a whole, this effect is not statistically significant, and so the deviance between NHI/TTH time series data and IBM outputs are not statistically different from each other. The apparent effect could be the result of stochastic fluctuations. The probability of obtaining this effect by chance is p = 0.252. This means the effect may be spurious and would generally not be considered statistically significant.

Supplementary Table 1. Details of Bayesian inference time series intervention analysis (Causal impact) for *Scenario I*.

```
Posterior inference {CausalImpact}

                         Average            Cumulative
Actual                   0.072              3.114
Prediction (s.d.)        0.074 (0.0018)     3.163 (0.0785)
95% CI                   [0.07, 0.077]      [3.01, 3.325]

Absolute effect (s.d.)   -0.0011 (0.0018)   -0.0486 (0.0785)
95% CI                   [-0.0049, 0.0025]  [-0.2112, 0.1071]

Relative effect (s.d.)   -1.5% (2.5%)       -1.5% (2.5%)
95% CI                   [-6.7%, 3.4%]      [-6.7%, 3.4%]

Posterior tail-area probability p:   0.25178
Posterior prob. of a causal effect:  75%
```

<u>Scenario II</u>

During the post-intervention period, the response variable had an average value of approx. 0.072. By contrast, in the absence of an intervention, we would have expected an average response of 0.086. The 95% interval of this counterfactual prediction is [0.082, 0.090]. Subtracting this prediction from the observed response yields an estimate of the causal effect the intervention had on the response variable. This effect is -0.013 with a 95% interval of [-0.017, -0.0096].

Summing up the individual data points during the post-intervention period, the response variable had an overall value of 3.11. By contrast, had the intervention not taken place, we would have expected a sum of 3.69. The 95% interval of this prediction is [3.53, 3.85]. The above results are given in terms of absolute numbers. In relative terms, the response variable showed a decrease of-15%. The 95% interval of this percentage is [-20%, -11%].

This means that the negative effect observed during the intervention period is statistically significant. The probability of obtaining this effect by chance is very small (Bayesian one-sided tail-area probability p = 0.001). This means the causal effect can be considered statistically significant.

Supplementary Table 2. Details of Bayesian inference time series intervention analysis (Causal impact) for *Scenario II*.

```
Posterior inference {CausalImpact}

                         Average            Cumulative
Actual                   0.072              3.114
Prediction (s.d.)        0.086 (0.0019)     3.685 (0.0818)
95% CI                   [0.082, 0.09]      [3.528, 3.85]

Absolute effect (s.d.)   -0.013 (0.0019)    -0.571 (0.0818)
95% CI                   [-0.017, -0.0096]  [-0.735, -0.4139]

Relative effect (s.d.)   -15% (2.2%)        -15% (2.2%)
95% CI                   [-20%, -11%]       [-20%, -11%]

Posterior tail-area probability p:   0.00102
Posterior prob. of a causal effect:  99.89848%
```

Supplementary Note 6: Synchrony between English Midlands and Wales

Quantifying the impact of annual testing introduction in Wales in 2010

Part I: Data

We have performed additional analysis calculating covariance matrixes of differences in NHI/TTH after testing interruption, between counties in Wales, English Midlands and English North regions. Our rationale was to examine the effect of testing on population synchrony and we thus compare counties in Wales with its most synchronised region (counties in Midlands of England) as well as Wales with its least synchronised region (counties in North England); (Fig. S6a; S6b). From December 2002 until December 2009 all three regions (Wales, Midlands, North) had the same testing policy. Since January 2010, Wales has at least annual testing and we therefore compared synchrony after testing interruption between the three regions but before the change in testing policy in Wales (Dec 2002 – Dec 2009; Fig. S6a) and after the change in testing policy in Wales (Jan 2010 – Sept 2015; Fig. S6b). These additional figures (Fig. S6c; S6d) show that after the introduction of at least annual testing in Wales, the magnitude of synchronisation between Wales and its most synchronised region, Midlands, is considerably lower than before (Fig. S6c; S6d). In addition, the magnitude of synchronisation between Wales and its least synchronised region, English North, is also lower than before (Fig. S6c; S6d). Contrastingly, this is not the case between English Midlands and North (Fig. S6c; S6d). These results show that the introduction of annual testing in Wales in 2010 had a strong effect in desynchronizing Welsh counties with counties in English Midlands, the region that was most synchronised with Wales.

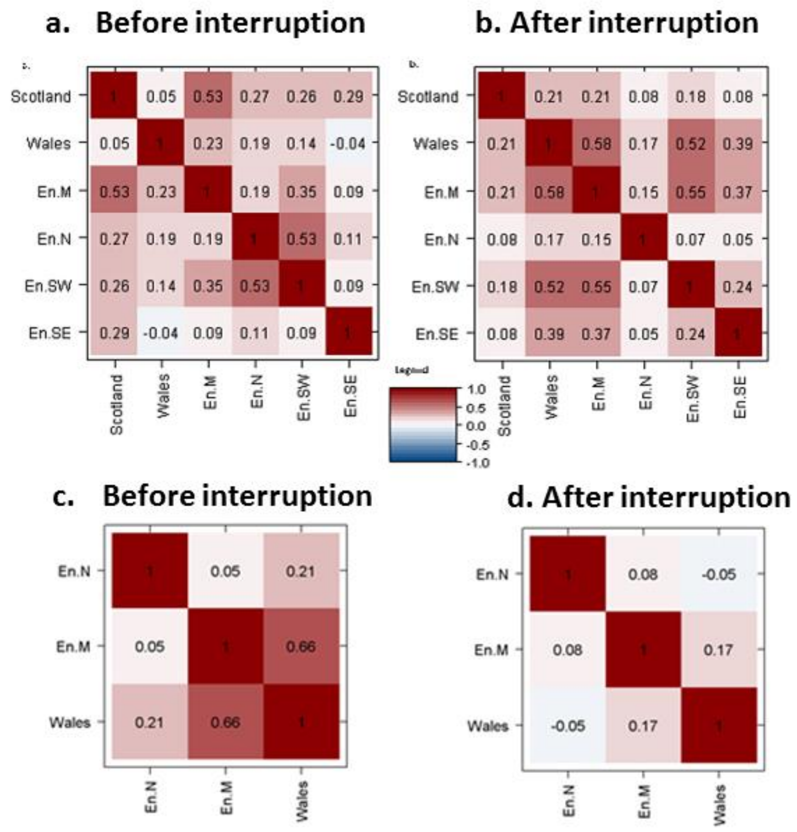

Supplementary Figure 7. Correlation matrices of NHI/TTH data between Wales, North England, English Midlands. a. All regions in GB counties aggregated per region before testing interruption. **b.** All GB counties aggregated per region after testing interruption. **c.** Wales, North England, and English Midlands counties. After testing interruption but before the change in testing policy in Wales (December 2002 to December 2009). **d.** Wales, North England, and English Midlands counties. After introducing at least annual testing in Wales (January 2010 to August 2016). Interruption data are discarded here. Wales was compared with Midlands because its most synchronized region and English North its least synchronized region in GB.

We initialised and run the model twice, once for Midlands and once for Wales, with all model parameters been identical except the initial percentage of infected herds and testing frequency. The model was run from Nov 2002 to Aug 2016 (after testing interruption).

For Midlands the model was initialised with an initial percentage of infected herds of 3.02% corresponding to the percentage of herds not bTB free in English Midlands in November 2002 (the first month after testing interruption) from the dataset [19]. This was achieved by dividing 'Herds not officially TB free' in the dataset by adding the total herds registered in Midlands in the same month [19]; [(505 / 16743) x 100 = 3.02%]. Testing frequency was initially implemented such as disease restricted herds were tested annually and the remaining herds every four years; this resulted in 435/16,806 x100 = 2.6% of herds were tested annually and the remaining tested every four years (currently and since 2013 annual testing is applied in high risk areas and also in edge areas but this was not applicable in 2002; there were also pockets of less frequent routine testing within the annual testing area at the time [20]). In January 2013 there were 12,559 herds registered of which 8,263 in high risk areas, 3,336 in edge areas and 960 in low risk areas. Thus sequentially testing was implemented in the model such as [(8,263 + 3,336 / 12,559) x 100 = 92% were tested annually and 8% were tested every four years. The model was run for 119 months corresponding to the post interruption period November 2002 – December 2012 with four year testing policy. Sequentially from month 120 (January 2013) to month August 2016 testing frequency was set to 92% annual and 8% of herds every four years.

For Wales the model was initialised with an initial percentage of infected herds of 2.61% corresponding to the percentage of infected herds in Wales in December 2002 derived by dividing 'Herds not officially TB free' by 'number of cattle herds registered' in the dataset; [(454 / 17407) x 100 = 2.61%). Testing frequency was initially implemented such as disease restricted herds were tested annually and the remaining herds every four years; this resulted in [(402/17635 x 100] = 2.3% of herds were tested annually and the remaining tested every four years. The model was run for 97

months corresponding to the post interruption period November 2002 – December 2009. Sequentially from month 96 (December 2009) to month August 2016 testing frequency was set to annual testing for al herds.

Results (in terms of NHI/TTH) from the IBM simulations calibrated for Midlands and Wales are plotted as empirical probability density functions using the identical methodology described in the section ‘Empirical Probability Distributions’ in the main text. These empirical probability density functions were compared with NHI/TTH from Wales and Midlands for the same period. These results plotted in Figures 4c, 4d, 4e, and 4f in the main document. These figures (4c- 4f) show that data and outputs from a mechanistic IBM model agree on the post annual testing introduction in Wales in 2010 desynchronization between counties of Wales and English Midlands. Wales are compared with Midlands because it was the most synchronized region with Wales.

The efficacy of annual testing introduced in Wales in 2010 is further enhancing the reduction of NHI/TTH as time evolves. Before the introduction of annual testing in Wales (before 2010) NHI/TTH had both larger mean values as well as high variance (brown colour). While the empirical probability density of NHI/TTH after the introduction of annual testing in 2010 has shifted towards lower values and the variance of these values is also lower (yellow colour), this result is even more pronounced during the last three years (2013-2016) indicating a consistency in reducing NHI/TTH mean and variability as evident from the figure below reconstructed with data from the full time series:

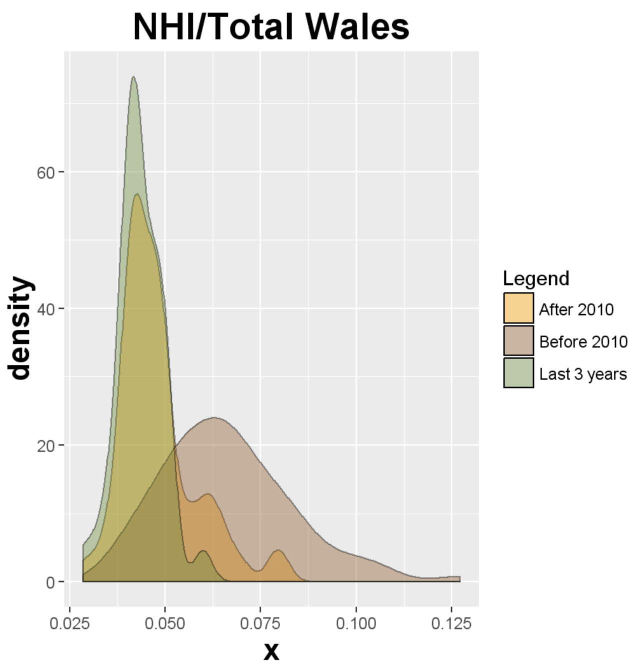

Supplementary Figure 8. Probability density plots in Wales. Empirical probability density plots of NHI/TTH in counties in Wales before 2010 (brown colour) after 2010 (yellow colour) and during the last three years (2013 – 2016). Annual testing interruption was introduced in Wales in 2010. The empirical probability distribution aggregated across all counties in wales was derived with the same procedure as the one described in the main text, Methods, ‘*Empirical Probability Distributions’*

Supplementary Note 7: Quantifying the potential deviance between disease Incidence and NHI/TTH

Throughout this study the NHI/TTH was used an index of bTB incidence. However, the NHI/TTH index refers to herds and not individuals and accounts for new herd incidents. In epidemiology a commonly used index is disease incidence. Incidence is the rate of new (or newly diagnosed) cases of the disease and would be reported as the number of new (bTB) cases occurring within a time unit (e.g. month or year), normalized by the fraction of the population at risk of developing the disease (e.g., per 1,000 cattle individuals). Obviously, the accuracy of incidence data depends upon the accuracy of diagnosis and reporting of the disease, which is not reported by the data publisher. Throughout this work NHI/TTH was used as an index of disease incidence, we sought to quantify the relationship between incidence at the level of individual cattle and NHI/TTH. Calculating incidence at a county scale to the level of individuals was not feasible with the current dataset format.

Using 'individual's-time' rather than just time handles situations where the amount of observation time differs between individuals, or when the population at risk varies with time. This measure is employed under the assumption that the incidence rate is constant over different periods of time. For example, an incidence rate of 7 per 1000 individual-years, 7 cases would be expected for 1000 herds observed for 1 year or 50 herds observed for 20 years.

Assume that in a sample population of e.g. 2000 cattle, at the beginning of the study (t=0) there are 250 bTB individual infected cattle cases. These cattle individuals are not counted as they cannot develop bTB again. After 12 months the cattle are surveyed again (t=12 months) and 200 new cases (infected cattle individuals) were recorded. After 46 months from the beginning of the study (t=46 months) 300 new infected cattle individuals (cases) were recorded.

To measure incidence one must take into account how many time units each individual contributed to the study, and when they developed bTB. When it is not known exactly when an individual develops the disease, epidemiologists frequently use the actuarial method, and assume it was developed at a half-way point between follow-ups. In this calculation:

At 12 months we found 200 new cases, so we assume they developed bTB at 6 months, thus contributing (200 * 6) =1200 cattle-months of disease-free life.

At 46 months we found 300 new cases. These individuals did not have bTB at 12 months, but did at 46 months, so we assume they were infected at [(46-12)/2 +12] = 29 months, thus contributing (300 * 29) = 8700 cattle-months of disease-free life. That is a total of (1,200 + 8,700) = 9900 cattle months so far.

We also need to account for the (3000-(250+200+300)) = 2250 individuals who did not develop bTB over the 46-month period, (2250 * 46) contributing 103500 cattle-months of disease-free life.

Taking the (200 + 300) = 500 new cases of infected cattle individuals and divide by (103500+1200+8700) cattle months of disease free life we get a 0.0044 or 4.4 infected cattle individuals per 1000 population per month.

Using the methodology described above we compared NHI/TTH from the dataset with bTB cattle incidence calculated from IBM outputs across all modelled scenarios. Specifically, for each month from January 1996 to August 2016 we compared the NHI/TTH with the corresponding IBM-derived incidence for the corresponding time step. NHI/TTH values were averaged across all counties for each time step. Incidence values were averaged across all IBM simulations for each time step. Doing so we sought to provide a (crude) estimate of the relationship between NHI/TTH and true incidence.

A linear regression fit between NHI/TTH as dependent variable and incidence as independent variable was conducted. All values were log_{10}-transformed prior to the regression.

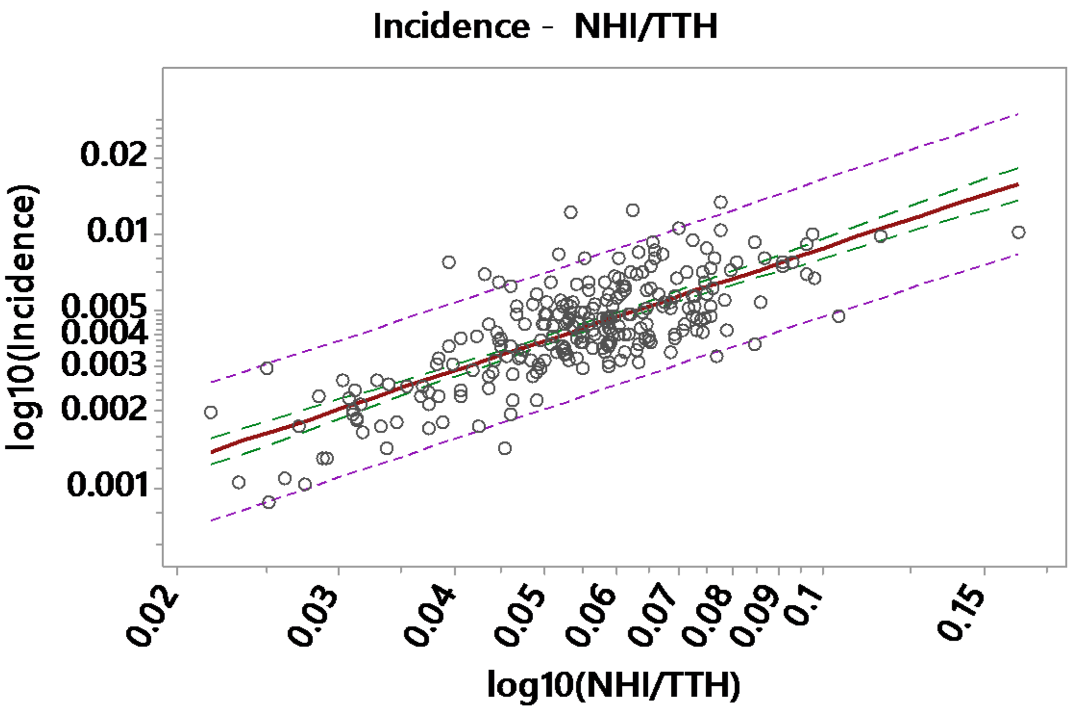

Supplementary Figure 9. Linear regression between log_{10}(incidence) and log_{10}(NHI)

```
Regression equation:
log10(Incidence) = - 0.8478 + 1.211 log10(NHI/TTH)
```

S = 0.136539 R^2 = 57.4%

Analysis of Variance

Source	DF	SS	MS	F	P
Regression	1	6.224	6.224	333.84	<0.001
Error	246	4.586	0.019		
Total	247	10.810			

The analysis indicated that NHI/TTH is at least one level of magnitude larger than incidence. Overall there is a good fit between NHI/TTH and incidence, however there are cases where NHI/TTH, an

index with few tests/cases, does not predict incidence, an index with many tests/cases. In order to account for this in the synchrony analysis (Figure 3 in the main text and Figures S4 and S6 in the supplement) only counties above the threshold were plotted (counties with at least six NHI months > 0, and counties with (NHI/TTH)> 0.3). In general, a high proportion of a large number of cases (incidence) is more likely to pose a threat that a high proportion of fewer cases (NHI/TTH). To that end the results reported here are likely to underestimate disease spread and synchrony in comparison to the results that would have been derived had this work used incidence in events of large numbers of cases.

Supplementary References

1. Guarte JM, Barrios EB. Estimation Under Purposive Sampling. Communications in Statistics - Simulation and Computation. 2006;35(2):277-84. doi: 10.1080/03610910600591610.
2. DEFRA. Annex - Background and methodology to the National Statistics on the Incidence of Tuberculosis (TB) in Cattle in Great Britain 2016. Available from: https://www.gov.uk/government/uploads/system/uploads/attachment_data/file/508001/bovinetb-annex-16mar16.pdf.
3. Moustakas A, Evans MR. Regional and temporal characteristics of bovine tuberculosis of cattle in Great Britain. Stoch Environ Res Risk Assess. 2016;30(3):989-1003. doi: 10.1007/s00477-015-1140-3.
4. Venables WN, Ripley BD. *Modern Applied Statistics with S. Fourth Edition*. Springer, New York. ISBN 0-387-95457-0. 2002.
5. R Development Core Team. R; A language and environment for statistical computing. Vienna, Austria: R Foundation for Statistical Computing; 2018.
6. Liu Y, Liang XS, Weisberg RH. Rectification of the Bias in the Wavelet Power Spectrum. Journal of Atmospheric and Oceanic Technology. 2007;24(12):2093-102. doi: 10.1175/2007jtecho511.1.
7. Veleda D, Montagne R, Araujo M. Cross-Wavelet Bias Corrected by Normalizing Scales. Journal of Atmospheric and Oceanic Technology. 2012;29(9):1401-8. doi: 10.1175/jtech-d-11-00140.1.
8. Cazelles B, Chavez M, Magny GCd, Guégan J-F, Hales S. Time-dependent spectral analysis of epidemiological time-series with wavelets. Journal of The Royal Society Interface. 2007;4(15):625-36. doi: 10.1098/rsif.2007.0212.
9. Grinsted A, Moore JC, Jevrejeva S. Application of the cross wavelet transform and wavelet coherence to geophysical time series. Nonlin Processes Geophys. 2004;11(5/6):561-6. doi: 10.5194/npg-11-561-2004.
10. Moustakas A, Evans M. Coupling models of cattle and farms with models of badgers for predicting the dynamics of bovine tuberculosis (TB). Stoch Environ Res Risk Assess. 2015;29(3):623-35. doi: 10.1007/s00477-014-1016-y.
11. Eurostat. Farm Structure Survey in the United Kingdom - 2007. http://epp.eurostat.ec.europa.eu/cache/ITY_OFFPUB/KS-SF-09-081/EN/KS-SF-09-081-EN.PDF: 2009.
12. FSA. Clean Beef Cattle for slaughter A guide for producers. Food Standards Agency Publications FSA/0951/1007 www.food.gov.uk/multimedia/pdfs/publication/cleanbeefsaf1007.pdf2007. Available from: www.food.gov.uk/multimedia/pdfs/publication/cleanbeefsaf1007.pdf.
13. Krebs JR, Anderson R, Clutton-Brock T, Morrison I, Young D, Donnelly CA, et al. Bovine tuberculosis in cattle and badgers. Report to the Rt Hon Dr Jack Cunningham MP by The Independent Scientific Review Group, London, 191 pages. London: 1997.
14. Conlan AJK, McKinley TJ, Karolemeas K, Pollock EB, Goodchild AV, Mitchell AP, et al. Estimating the Hidden Burden of Bovine Tuberculosis in Great Britain. PLoS Comput Biol. 2012;8(10):e1002730. doi: 10.1371/journal.pcbi.1002730.
15. Gilbert M, Mitchell A, Bourn D, Mawdsley J, Clifton-Hadley RS, Wint W. Cattle movements and bovine tuberculosis in Great Britain. Nature. 2005;435:491-6. doi: 10.1038/nature03548.
16. Claridge J, Diggle P, McCann CM, Mulcahy G, Flynn R, McNair J, et al. Fasciola hepatica is associated with the failure to detect bovine tuberculosis in dairy cattle. Nature Comms. 2012;3. doi: 10.1038/ncomms1840.
17. Brodersen KH, Gallusser F, Koehler J, Remy N, Scott SL. Inferring causal impact using Bayesian structural time-series models. The Annals of Applied Statistics. 2015;9(1):247-74.

18. Moustakas A. Assessing the predictive causality of individual based models using Bayesian inference intervention analysis: an application in epidemiology. Stoch Environ Res Risk Assess. 2018:doi: 10.1007/s00477-018-1520-6. doi: 10.1007/s00477-018-1520-6.
19. DEFRA. Monthly publication of National Statistics on the Incidence of Tuberculosis (TB) in Cattle to end September 2015 for Great Britain. 2015. Available from: https://www.gov.uk/government/statistics/incidence-of-tuberculosis-tb-in-cattle-in-great-britain.
20. DEFRA. 2015 TB Testing Intervals Policy (England). https://www.gov.uk/government/uploads/system/uploads/attachment_data/file/503615/tb-test-intervals-eng-policy.pdf: 2015.
