# Supplementary material for: Abrupt events and population synchrony in the dynamics of Bovine Tuberculosis
Source: Nat Commun. 2018 Jul 19;9:2821. doi: 10.1038/s41467-018-04915-0 (PMC6053421; doi:10.1038/s41467-018-04915-0)
Supplement: Supplementary file 3 — Description of Additional Supplementary Files [file 41467_2018_4915_MOESM3_ESM.pdf]

Description of Additional Supplementary Files:

Supplementary Data 1:

Full dataset of NHI and TTH per county in GB. The file contains the full dataset used in the analysis reported here. The dataset contains NHI (nhi) and TTH (tth) per county and risk assessment status. The time step is one month. The (x, y) columns indicate the center of each county. Counties that were clustered at two risk statuses appear twice in the dataset with the corresponding values for each risk section of the county as well as the center of the risk section of the county.
